# Mobile Phone Text Messaging for Tobacco Risk Communication Among Young Adult Community College Students: Protocol and Baseline Overview for a Randomized Controlled Trial

**DOI:** 10.2196/10977

**Published:** 2018-10-15

**Authors:** Alexander V Prokhorov, Georges Elias Khalil, Karen Sue Calabro, Tamara Costello Machado, Sophia Russell, Katarzyna W Czerniak, Gabrielle C Botello, Minxing Chen, Adriana Perez, Damon J Vidrine, Cheryl L Perry

**Affiliations:** 1 Department of Behavioral Science MD Anderson Cancer Center The University of Texas Houston, TX United States; 2 Department of Biostatistics MD Anderson Cancer Center The University of Texas Houston, TX United States; 3 Department of Biostatistics and Data Science School of Public Health in Austin The University of Texas Health Science Center at Houston (UTHealth) Austin, TX United States; 4 Department of Family and Preventive Medicine The University of Oklahoma Health Sciences Center The University of Oklahoma Oklahoma City, OK United States; 5 School of Public Health in Austin The University of Texas Health Science Center at Houston (UTHealth) Austin, TX United States

**Keywords:** tobacco use, risk, perception, text messaging, young adult

## Abstract

**Background:**

Community-college students are at high risk for tobacco use. Because the use of mobile phone text messaging is nearly ubiquitous today, short message service (SMS) may be an effective strategy for tobacco risk communication in this population. Little is known, however, concerning the message structure significantly influencing perceived tobacco risk.

**Objective:**

We aim to outline the rationale and design of Project Debunk, a randomized trial comparing the effects of different SMS text message structures.

**Methods:**

We conducted a 6-month randomized trial comparing 8 arms, based on the combination of the 3 message structures delivered to young adults in a 2×2×2 study design: framing (gain-framed or loss-framed), depth (simple or complex), and appeal (emotional or rational). Participants were invited to participate from 3 community colleges in Houston from September 2016 to July 2017. Participants were randomized to 1 arm and received text messages in 2 separate campaigns. Each campaign consisted of 2 text messages per day for 30 days. Perceived tobacco risk was assessed at baseline, 2 months after the first campaign, and 2 months after the second campaign. We assessed the perceived risk of using conventional products (eg, combustible cigarettes) and new and emerging products (eg, electronic cigarettes). The validity of message structures was assessed weekly for each campaign. A 1-week follow-up assessment was also conducted to understand immediate reactions from participants.

**Results:**

We completed data collection for the baseline survey on a rolling basis during this time and assessed the validity of the message structure after 1 week of SMS text messages. For the entire sample (N=636), the average age was 20.92 years (SD 2.52), about two-thirds were male (430/636, 67.6%), and most were black or African American (259/636, 40.7%) or white (236/636, 37.1%). After 1 week of receiving text messages, the following was noted: (a) loss-framed messages were more likely to be perceived as presenting a loss than gain-framed messages (*F*_7,522_=13.13, *P*<.001), (b) complex messages were perceived to be more complex than simple messages (*F*_7,520_=2.04, *P*=.05), and (c) emotional messages were perceived to be more emotionally involving than rational messages (*F*_7,520_=6.46, *P*<.001).

**Conclusions:**

This study confirms that the recruitment, randomization, and message composition have been successfully implemented. Further analyses will identify specific types of messages that are more effective than others in increasing the perceived risk of tobacco use. If our results suggest that any of the 8 specific message structures are more effective for helping young adults understand tobacco risk, this would provide evidence to include such messages as part of a larger technology-based campaign such as mobile phone apps, entertainment-based campaigns, and social media.

**Trial Registration:**

ClinicalTrials.gov NCT03457480; https://clinicaltrials.gov/ct2/show/NCT03457480 (Archived by WebCite at http://www.webcitation.org/6ykd4IIap)

**Registered Report Identifier:**

RR1-10.2196/10977

## Introduction

### Background

Almost 14% of young adults are currently using cigarettes and 27% have used electronic cigarettes, one of the many new and emerging tobacco products (NETP) [1]. Young adults perceive NETPs such as electronic cigarettes (e-cigarettes) and hookah (ie, waterpipes) as safer ways to enjoy nicotine than conventional products [2-4]. Reduced risk perception has led to uninformed choices among young adults [5], including experimentation with multiple tobacco products, alcohol, and other substances [6-8]. Indicators of socioeconomic disadvantage such as low educational attainment and income status are predictors of tobacco use [9]. In particular, young adults in community college represent an underserved population more susceptible to tobacco use than young adults attending universities or 4-year colleges [10-12].

Following bans on traditional advertising for tobacco, protobacco marketing began to make effective use of modern advertising through social and mobile media channels to reduce the risk perception and promote misinformation about tobacco among young adults [13,14]. Currently, tobacco companies make effective expenditures on product discounts, point-of-sale advertising, direct mail advertising, e-marketing, and social media [15-20]. In addition, with 96% of young adults owning a smartphone, tobacco companies depend on mobile phone strategies for marketing [21]. Tobacco product demonstrations are featured on industry-sponsored websites, and invitations to join Web-based social interactions are encouraged [22-25]. More than 49 protobacco smartphone apps have been identified in app stores under *kids* and *games* categories [26]. As a result, there is a clear need for efforts to respond to protobacco marketing by communicating about tobacco risk to young adults, as delineated by the educational mission and research priorities of the United States Food and Drug Administration [27,28].

The use of mobile health (mHealth) SMS (short message service) text messaging may be an effective strategy for tobacco risk communication to young adults. In the United States, 95% of mobile phones are capable of receiving text messages and 96% of the young adults own mobile phones, indicating this is a highly feasible method for transmitting information to this population [21,29]. Although text messaging programs have been implemented for preventive behavioral interventions, including smoking cessation, no published accounts have applied text messaging to communicate about tobacco risk to young adults [30-34]. To the best of our knowledge, this study is the first to examine different styles of mobile phone text messages for tobacco risk communication. Once the most impactful text messages have been identified, they can subsequently be introduced into an advanced digital intervention that can counteract protobacco marketing.

In the United States, a majority of young adults have smartphones, with more advanced text messaging capabilities (eg, WhatsApp). However, it is pertinent to conduct an evaluation of text messages for risk communication, through traditional text messaging. SMS text messaging ensures that all participants are capable of receiving text messages regardless of a smartphone ownership. In addition, traditional text messaging ensures that all participants receive the messages in the same format, thereby allowing a homogeneous exposure to the intervention content and a more reliable evaluation. Such an evaluation will shed light on how the messages perform. If a set of text messages shows effectiveness, then it can readily be implemented among young adult communities outside the United States, where smartphone capabilities may be limited.

### Theoretical Framework

We have designed different types of messages based on 3 main structures: framing (gain-framed or loss-framed messages), depth (ie, simple or complex messages), and appeal (ie, emotional or rational messages) [35-38]. For framing, gain-framed messages describe the benefits of quitting or avoiding tobacco use, whereas loss-framed messages emphasize the disadvantages of use [39-41]. In the context of message depth, both complex grammatical structures and longer words have been applied to shape message complexity [42-45]. In terms of appeal, researchers have developed emotional SMS text messages by introducing emotional words (eg, *happy* and *angry*) [46-48], paralinguistic cues such as vocal spelling (eg, *weeeell* and *soooo*), and emotional icons (eg, “:-)” for a happy face) [49]. Most research has been in gain-framed versus loss-framed text messages [50]. Some literature, predominantly in advertising and promotion, has been dedicated to emotional versus rational appeal [47]. Virtually nothing has been reported on simple versus complex messages in the health risk domain.

The effectiveness of different message characteristics in driving risk communication outcomes stems from the elaboration likelihood model (ELM) [51,52]. The ELM explains motivation of the individual to engage in information processing. Individuals expending more mental or cognitive effort processing messages tend to formulate stronger attitudes toward an issue and deeper understanding—a desirable attribute for conveying tobacco risk information to the public. One of the basic constructs in the model concerns the degree of cognitive efforts expended and involvement that people use to engage with message content. The ELM posits that individuals can engage in either the central or peripheral processing of health information. Central processing involves attention to message content (eg, complex and rational messages; [53]), whereas peripheral processing involves attention to more peripheral cues such as affect or emotions in developing attitudes toward the message [54]. In the context of risk communication, researchers have not yet presented a theoretical framework supporting certain message types over others with respect to increasing perceived risk. For instance, in the context of message-framing, theoretical frameworks (eg, the prospect theory) do not support a specific framing over another with respect to increasing perceived risk [55]. Instead, such frameworks posit that gain and loss framing can have an effect on health behavior depending on whether the individual is risk-aversive or risk-taking. As a result, theoretical frameworks on message framing have not yet examined perceived risk as the end outcome. In addition, results from previous meta-analyses of relevant research have not been able to favor one message style over another, with respect to health outcomes [56-58]. As a result, it is essential to explore the effect of different message characteristics on perceived risk.

### Research Objectives

The primary objective of this study was to conduct exploratory analyses to identify the most effective types of text messages that inform about the harms of tobacco use among young adults in community college. This research protocol outlines the rationale and design of Project Debunk, a community-based randomized trial (peer-reviewed and funded; Multimedia Appendix 1). Project Debunk compares the effects of different structures of text messages delivered to young adults in community college, with the overarching goal of setting the stage for a larger mobile phone text messaging campaign in the future. The protocol presents baseline data from the trial and assesses the validity of the message structures after 1 week of SMS text message exposure.

## Methods

### Study Design

Project Debunk has gathered data in the following 2 phases: (1) qualitative research for text message development and (2) a randomized trial. This research protocol briefly describes the methods used for the message-development phase and outlines the detailed information about the trial phase at baseline and 1 week after message exposure (ie, the intervention).

**Figure 1:**
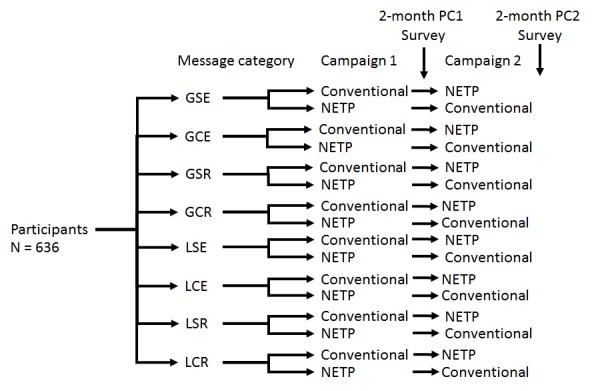
Study randomization flowchart. Conventional indicates conventional tobacco products including cigarettes, cigars, smokeless; NETP indicates new and emerging tobacco products, including snus, hookah, and e-cigarettes. In this study design, there is a break of one week between post-campaign 1 survey and campaign 2. GSE: gain-framed, simple emotional; GCE: gain-framed, complex, emotional; GSR: gain-framed, simple, rational; GCR: gain-framed, complex, rational; LSE: loss-framed, simple, emotional; LCE: loss-framed, complex, emotional; LSR, loss-framed, simple, rational; LCR: loss-framed, complex, rational; PC1: post-campaign 1; PC2: post-campaign 2.

In design, the trial is being conducted as a 6-month-long randomized trial comparing 8 arms, based on the combination of the 3 message structures: framing, depth, and appeal (Figure 1). Participants are randomly assigned to one of the 8 arms. They are receiving text messages in 2 separate waves or campaigns. Each campaign consists of 2 text messages per day for 30 days (ie, 60 text messages). The 2 campaigns are 2 months and 1 week apart.

Allowing for a crossover design, participants within each of the 8 arms are randomly divided into 2 groups: group 1 is receiving text messages about conventional tobacco products during campaign 1 and then about NETP during campaign 2. Group 2 is receiving text messages about NETP during campaign 1 and then about conventional tobacco during campaign 2. This crossover design was advised by the Tobacco Center of Regulatory Science on Youth and Young Adults (TX TCORS) Scientific Steering Committee, as it will allow us to explore potential differences between the 2 categories of products within and between participants, with respect to their perceived risk of tobacco use. Data collection for the trial is being conducted at baseline, 2 months post campaign 1 (PC1), 2 months post campaign 2 (PC2), weekly throughout each campaign (a weekly manipulation check assessment), and 7 days after each campaign.

### Population

Eligibility criteria for the trial included the following: aged 18 to 25 years, enrolled in community college, using mobile phone text-messaging features on a regular basis, willing to provide their phone number, capable of receiving text messages from our text messaging system, able to read and speak English, and accept to provide a signature on a written informed consent form. The age range of 18 to 25 years was chosen to define emerging young adulthood, as recommended by the National Research Council of the Institute of Medicine in the United States [59]. Three community college campuses from the Houston Community College (HCC) system were targeted for recruitment. Students attending the HCC system are 58% female and have a mean age of 25.6 years. Their racial or ethnic profile is as follows: 30.2% African American, 14.6% Asian American, 14.2% white, 36.9% Hispanic, and 4.2% other [60]. The 3 community colleges were selected based on their ethnically diverse student population and their proximity to our research institution. In addition, we have an existing research relationship with such institutions. All methods and procedures used in the project have been approved by the Institutional Review Board of Ethics of the University of Texas MD Anderson Cancer Center (2014-0474), as well as the HCC System Institutional Review Board.

### Recruitment and Enrollment

Recruitment took place at each of the participating HCC campuses from September 2016 to July 2017. We set up recruitment stations or booths equipped with a highly visible logo of the research institution. Printed materials (eg, posters and fliers) announcing the study were displayed in common areas such as student lounges. During participant recruitment at each campus, the research staff explained the purpose of the study to students and answered their questions. Students interested in the trial were screened for eligibility. Subsequently, eligible students provided informed consent to participate in the trial.

Following consent, participants completed a 20-min self-administered baseline survey on their personal mobile phones. This method of enrollment has yielded relatively high recruitment rates (80.1%) during our previous research activities with community college students [61]. Recruitment continued until a sample size of 645 participants was reached. In total, 9 participants were not eligible for the study (over the age of 25 years), so they were dropped, reaching a sample of 636 participants. Up to 6 follow-up reminders were sent via phone and email to remind participants to complete follow-up surveys to progress through the study.

### Text Message Interventions for Each Group

From January 2014 to August 2015, our research team from the TX TCORS developed a library of text messages, considering previous scientific literature, developments in social media related to tobacco use, and common terminology. Collectively, the research team has extensive experience in tobacco cessation and prevention, public health, health communication, psychology, and creative writing. Text message design also involved focus group discussions conducted among community college students [62].

Ultimately, our team generated 976 text messages that communicate the risks of tobacco use to college students, both users and nonusers. The messages were developed according to a combination of the 3 structures described above (framing, depth, and appeal), resulting in the following 8 categories:

- Complex, gain-framed, emotional (CGE)
- Complex, gain-framed, rational (CGR)
- Complex, loss-framed, emotional (CLE)
- Complex, loss-framed, rational (CLR)
- Simple, gain-framed, emotional (SGE)
- Simple, gain-framed, rational (SGR)
- Simple, loss-framed, emotional (SLE)
- Simple, loss-framed, rational (SLR)

In addition, for each category, messages were developed to communicate about the harm of conventional tobacco products and NETPs. Messages describing conventional products included information about combustible cigarettes, variants of cigars, cigarillos, and pipes. Messages about NETP included information about e-cigarettes (including other vaping devices), snus, and hookah. Examples of text messages are presented in Multimedia Appendix 2. Experts and students reviewed and rated each message. For validation of message categories, agreement needed to be ≥70% between experts and students for all the 3 message structures. Further validation of message categories was conducted using a linguistic inquiry and word-counting library designed to count words under specific themes (eg, emotional words) [63].

### Randomization and Blinding

This is a double-blind study. Following screening and consent, members of our research staff provided participants with a study identification number and a link to the baseline survey to the mobile phone of each participant. This procedure confirmed that the participant’s device fully met the needs of the study. Following the baseline survey, participants were assigned to one of the 8 arms following a computer-generated randomization list using a resource called assessment, intervention, and measurement (AIM). AIM is a centralized repository at the MD Anderson Cancer Center, managed by a team of experts in the science of collecting and managing participant-reported outcomes. The allocation sequence was generated by the AIM system and automatically sent text messages based on allocation, ensuring that our research team is blind to the allocation of each participant. The allocation sequence is password protected and accessible only to nonresearch staff responsible for the AIM system.

### Data Collection

**Figure 2:**
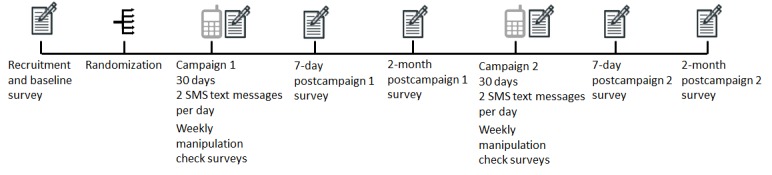
Data collection procedure for the study. SMS: short message service.

Figure 2 depicts how data are collected for the study. Data collection took place at baseline and will continue at the end of each week throughout campaign 1 and campaign 2 of text message dissemination, as well as 7 days PC1, 7 days PC2, 2 months PC1, and 2 months PC2. Participants will provide data through Web-based surveys received through mobile phones.

We developed the surveys with skip patterns to minimize the burden on participants. Using mobile phones from different brands and data carriers, the research team pretested the delivery of surveys and text messages with the assistance of experts in the AIM system (a team of computer scientists and bioinformaticians). This pretesting allowed us to ensure that the surveys and text messages are reachable and readable regardless of the mobile phone or data carrier. We conducted the pretesting initially with our immediate staff and research team. Afterwards, we extended to other staff in one of our departments (Department of Behavioral Science). We conducted an iterative process such that each time an issue was identified by survey testers, it was rectified. Pretesting continued until no issues were reported.

Data collection from the baseline survey ended in July 2017. At the end of each week throughout SMS text message exposure in campaign 1 and campaign 2, participants will complete a manipulation check survey. This weekly manipulation check will ensure that the 8 arms of the study differ with respect to unique features such as perceived emotional level, complexity of the text messages, and framing type. Data collection from the manipulation check survey for the first week of campaign 1 ended in October 2017. Participants will receive a survey regarding their immediate experience with the text messages 7 days PC1 and 7 days PC2. Finally, 2 months PC1 and 2 months PC2, participants will receive a follow-up survey that includes tobacco-related outcome measures.

### Survey Measures

All survey measures have been previously tested and validated, with some adaptations (further outlined below). All measures are assessed through Web-based closed surveys. We adhered to the Checklist for Reporting Results of Internet E-Surveys (Multimedia Appendix 3). This checklist will be reported once the study is completed, with the main outcomes of the trial. This paper presents data from the baseline survey and the first weekly manipulation check survey. A detailed description of the main measures and Cronbach alpha values for available data are reported in Multimedia Appendix 4.

#### Baseline Survey

The baseline survey data for the trial have been collected. With 97 items, baseline information included sociodemographic data such as age, gender, ethnicity, educational attainment, and income [64]. In addition, the baseline survey included questions about factors that may predict perceived risk and tobacco use: mental health status [65], marijuana and alcohol use [64], receptivity to receiving text messages [66], tendency to seek information about tobacco [67], number of friends using tobacco [68], secondhand smoke at home [64], mental health [69], prevention-focus level [70], sensation-seeking level [71], and numeracy ability [72].

#### Follow-Up Surveys

Follow-up surveys for the trial are ongoing. Weekly manipulation check surveys will assess perceptions of participants about text messages received in the previous week. The perceived message characteristics to be assessed include loss framing [73], message complexity level [74], emotional level of messages [50], credibility [75], message enjoyment [76,77], relevance [78], and message readability.

Surveys completed by participants 7 days after each of the 2 campaigns will assess self-reported attention to the text messages [79], emotional involvement [79], thought provocation [80], motivation to discuss the messages with others [81,82], and recall of actual discussions with others about the messages and tobacco [82].

Two months PC1 and 2 months PC2, we will measure perceived risk of using each tobacco product as the main outcome [83]. As secondary outcomes, we will also measure the status and frequency of tobacco use [84], susceptibility to use tobacco products among nonusers (ie, likelihood to initiate use at some point in the future) [85], perceived addictiveness of products [4], perceived popularity of tobacco use [4], and perceived benefits of tobacco use [86].

### Compensation

Participants who complete all survey assessments will be compensated a total of US $135. They received a US $25 gift card for completing the baseline survey and will receive a US $25 gift card for completing each of the surveys administered at 2 months after the campaigns. They will also receive a US $10 gift card for completing each of the surveys administered 7 days after the campaigns and a US $5 gift card for each of the 8 weekly manipulation check surveys throughout the 2 campaigns.

### Attrition and Compliance

To the best of our knowledge, no study examining the effects of different communication styles on risk perception among young adults is currently available. On the basis of the results of a study with community college students by Prokhorov and colleagues [61], we expect an acceptable retention rate (beyond 70%) and high compliance (ie, a self-report of paying attention to and reading most or all of the text messages, with a score of 4 or higher out of 5 on message attention).

### Sample Size Determination

For sample size determination, we conducted a power calculation using the outcome of change in perceived risk of cigarette smoking from baseline to 2 months after each of the campaigns. The 8 study arms define a 2×2×2 analysis of variance (ANOVA) factorial design. Assuming a balanced design in each of the 8 study arms for the change in perceived risk, with n=70 per arm, we have at least 80% power to detect an effect size of 0.12 in a fixed-effects ANOVA. A total of 560 participants are needed to provide 70 participants per study arm at 2 months PC2, with complete measurements at baseline. We assume 11% attrition between the assessment at 7 days after the program and 2 months after the program and 1.5% attrition between baseline and the assessment at 2 months PC2 (ie, a total of 12.5% attrition). As a result, our retention rate is expected to be 87.5%. This assumes 640 participants randomized to 8 study arms. This sample size was calculated using PASS 2005 (NCSS, LP).

### Current Data Analysis

For the currently available baseline data, we used descriptive statistics to summarize sociodemographic characteristics (eg, age, gender, and race), tobacco-related characteristics (tobacco use and number of friends who use tobacco), and primary psychosocial health outcomes (ie, perceived risk of using each tobacco product).

Using the currently available data from the first weekly manipulation check survey, we checked to make sure that the message structures were perceived by participants as intended. Using one-way ANOVA, we examined study arm differences in perceived message loss framing, complexity level, emotional level, credibility, message enjoyment, perceived message relevance, and perceived message readability. STATA version 14 statistical software was used for data analysis.

### Planned Data Analysis

Once the trial is complete, we plan to conduct an exploratory analysis to identify which combination of message characteristics (ie, depth, framing, and appeal) most increases perceived risk of using each type of tobacco product. This analysis is exploratory because to date no theoretical framework or empirical evidence has been presented that demonstrates the importance of one message structure over another in the context of tobacco risk communication. We will first conduct, a series of 8 repeated-measures mixed-effects models for each type of tobacco product with the interaction effect (group [one combination vs all other combinations] × time [baseline, 2 months PC1, and 2 months PC2]) to predict perceived risk of using the product. These models will control for past 30-day use of the product at baseline and the crossover group assignment. In addition to the main outcomes analyses, we plan to conduct several moderation analyses, including the examination of different groups such as gender, race, mental health status, and personality types. This analysis will allow us to check if different types of individuals may respond differently to certain structures of text messages. Repeated-measures mixed-effect models with interaction effects will be conducted. For all data analysis, *P*<.05 is considered statistically significant. We will use STATA version 14 software (StataCorp LLC) for all analyses.

### Ethics and Participant Safety

Project Debunk has received full approval from the Research Ethics Board of The University of Texas MD Anderson Cancer Center in Houston, Texas, and it has undergone a local institutional scientific review. To the best of our knowledge, Project Debunk does not pose any significant risks to the physical and psychological safety of participants. Identities of the participants have been coded and only the research team has access to a master list that links names and study codes. This list is kept in a locked file cabinet. Demographic data and assessments of text messages will be stored on secure servers within the institution. Only aggregate data will be reported. We have obtained a Certificate of Confidentiality from the federal government, which will help to protect the privacy of research participants. The certificate protects against the involuntary release of information about participants collected during the course of covered studies.

## Results

### Status of Results

Data collection is currently underway. Data analysis of change in the main outcomes and writing of the manuscript are expected to be completed in the summer of 2018. We highlight below some of the main baseline findings regarding the study population and measures.

### Sociodemographic Characteristics

Table 1 presents sociodemographic characteristics of the respondents. For the entire sample (n=636), the average age was 20.78 years (SD 2.18), about two-thirds (430/636, 67.6%) were male, and most were black or African American (259/636, 40.7%) or white (236/636, 37.1%). With respect to ethnicity, 36.3% (231/636) of participants were Hispanic or Latino. The study arms did not differ in terms of sociodemographic characteristics or mental health status, economic status, planned education level, numeracy ability, prevention-focus level, receptivity to receiving text messages, or sensation-seeking level (Table 1).

### Tobacco-Related Characteristics

Tobacco-related characteristics of the respondents are presented in Table 2. Of the entire sample, at least once in their lifetime, 45.1% (287/636) have ever used cigarettes, 32.4% (206/636) have ever used cigars, 55.7% (354/636) have ever used hookah, and 26.9% (171/636) have ever used e-cigarettes. In addition, 25.3% (161/636) have ever used marijuana, 47.2% (300/636) have ever used more than one tobacco product, and 43.2% (275/636) have ever used both marijuana and tobacco products.

Among nonusers, 13.4% (47/351) were found to be susceptible to smoking cigarettes, 24.3% (109/449) were susceptible to smoking cigars, 30.4% (86/283) were susceptible to using hookah, and 24.1% (106/440) were susceptible to using e-cigarettes. At baseline, no significant differences between the 8 groups were found with respect to all such tobacco-related characteristics (Table 2).

### Manipulation Checks

We first checked to ensure that the messages were perceived by participants as intended after the first week of message exposure (Table 3). Out of 636 participants, 530 (530/636, 83.3%) completed the manipulation check survey. Compared with gain-framed messages, loss-framed messages were significantly more likely to be perceived as presenting a loss, *F*_7,522_=13.13, *P*<.001. Groups receiving CLE, CLR, SLE, and SLR text messages scored higher on perceived message framing as loss than that of groups receiving CGE, CGR, SGE, or SGR text messages. Complex messages were perceived to be significantly more complex than that of simple messages, *F*_7,520_=2.04, *P*=.05. Groups receiving CLE, CLR, CGE, and CGR messages scored higher on perceived message complexity than that of groups receiving SLE, SLR, SGE, or SGR messages. Emotional messages were perceived to be significantly more emotionally involving than rational messages, *F*_7,520_=6.46, *P*<.001. The groups receiving CLE, SLE, CGE, and SGE messages scored higher on the perceived emotional level of their messages than did the groups receiving CLR, CGR, SLR, or SGR messages (Table 3).

We also checked to make sure that the health messages were consistently perceived as credible (Table 3). As expected, there was no significant difference among the treatment arms with regard to the perceived credibility of message content (*F*_7,520_=1.70, *P*=.10). The total mean score on SMS text message credibility was 7.57 (SD 2.01) on an 8-point scale. This confirms that all text message interventions were perceived to be credible sources of information related to tobacco. Similarly, as shown in Table 3, the treatment arms did not differ with regard to the enjoyment of the messages (*F*_7,520_=0.41, *P*=.90), perceived message relevance (*F*_7,517_=1.04, *P*=.40), or perceived message readability (*F*_7,517_=0.34, *P*=.94).

### Baseline Treatment Arm Differences in Outcome Measures

At baseline, there were no significant differences among the treatment arms with respect to our risk communication variables: perceived risk of using each tobacco product, perceived personal and general benefits of e-cigarettes, perceived addictiveness of products, or perceived popularity of tobacco use (Table 4).

**Table 1:** Baseline sociodemographic characteristics for the total sample and by treatment arm.

Characteristics		Statistics^a,b,c^								
		Total	CGE^d^	CGR^e^	CLE^f^	CLR^g^	SGE^h^	SGR^i^	SLE^j^	SLR^k^
Gender at birth (men), n (%)		430 (67.6)	53 (65.4)	52 (67.5)	54 (74.0)	60 (74.1)	56 (65.1)	56 (70.9)	44 (57.1)	55 (67.1)
**Race, n (%)**										
	Hispanic or Latino ethnicity	231 (36.3)	31 (38.3)	29 (37.7)	24 (32.9)	28 (34.6)	30 (34.9)	34 (43.0)	22 (28.6)	33 (40.2)
	Asian	99 (15.6)	12 (14.8)	15 (19.5)	13 (17.8)	9 (11.1)	14 (16.3)	7 (8.9)	16 (20.8)	13 (15.9)
	Black or African American	259 (40.7)	34 (42.0)	28 (36.4)	33 (45.2)	39 (48.1)	33 (38.4)	30 (38.0)	31 (40.3)	31 (37.8)
	White	236 (37.1)	31 (38.3)	29 (37.7)	21 (28.8)	29 (35.8)	36 (41.9)	31 (39.2)	24 (31.2)	35 (42.7)
	Other	42 (6.6)	4 (4.9)	5 (6.5)	6 (8.2)	4 (4.9)	3 (3.5)	11 (13.9)	6 (7.8)	3 (3.7)
Have children, n (%)		58 (9.1)	7 (8.6)	8 (10.4)	8 (11.0)	5 (6.2)	5 (5.8)	9 (11.4)	9 (11.7)	7 (8.5)
Age (years), mean (SD)		20.92 (2.52)	20.53 (2.21)	21.03 (2.10)	20.75 (2.10)	20.89 (2.30)	20.55 (2.06)	20.97 (2.25)	20.86 (2.22)	20.66 (2.22)
Mental health status, mean (SD)		67.33 (19.18)	67.80 (18.01)	64.83 (18.80)	68.11 (19.16)	69.43 (19.32)	67.53 (18.74)	68.30 (19.02)	67.17 (20.42)	65.46 (20.33)
Economic status, mean (SD)		2.77 (0.92)	2.67 (0.96)	2.79 (0.96)	2.74 (0.96)	2.68 (0.93)	2.84 (0.89)	2.96 (0.81)	2.77 (0.97)	2.71 (0.90)
Planned education level, mean (SD)		3.67 (1.15)	3.77 (1.10)	3.75 (1.05)	3.53 (1.28)	3.81 (1.16)	3.66 (1.06)	3.72 (1.09)	3.52 (1.25)	3.61 (1.24)
Numeracy ability, mean (SD)		5.35 (1.81)	5.41 (1.61)	5.51 (1.82)	5.29 (2.00)	5.15 (2.04)	5.56 (1.69)	5.20 (1.86)	5.39 (1.73)	5.27 (1.74)
Prevention-focus level, mean (SD)		2.81 (0.69)	2.73 (0.71)	2.90 (0.73)	2.86 (0.67)	2.77 (0.72)	2.80 (0.70)	2.80 (0.64)	2.79 (0.72)	2.82 (0.68)
Receptivity to receiving text messages, mean (SD)		0.92 (0.15)	0.94 (0.12)	0.90 (0.22)	0.90 (0.15)	0.89 (0.18)	0.92 (0.13)	0.95 (0.11)	0.94 (0.10)	0.93 (0.14)
Sensation-seeking level, mean (SD)		3.50 (0.83)	3.50 (0.73)	3.47 (0.85)	3.34 (0.91)	3.45 (0.86)	3.56 (0.78)	3.60 (0.84)	3.51 (0.85)	3.55 (0.81)

^a^Missing values are not presented in this table.

^b^Participants were randomized to one of the 8 treatment arms, describing the type of messages.

^c^Proportions in subsample and percentage are presented for categorical variables, and the mean with SD are presented for continuous variables.

^d^CGE: complex, gain-framed, emotional.

^e^CGR: complex, gain-framed, rational.

^f^CLE: complex, loss-framed, emotional.

^g^CLR: complex, loss-framed, rational.

^h^SGE: simple, gain-framed, emotional.

^i^SGR: simple, gain-framed, rational.

^j^SLE: simple, loss-framed, emotional.

^k^SLR: simple, loss-framed, rational.

**Table 2:** Tobacco-related characteristics for the total sample and by the group at baseline.

Substance use^a^		N^b^ (%)								
		Total	CGE^c^	CGR^d^	CLE^e^	CLR^f^	SGE^g^	SGR^h^	SLE^i^	SLR^j^
**Cigarettes**										
	Ever	287 (45.1)	37 (45.7)	34 (44.2)	28 (38.4)	35 (43.2)	43 (50.0)	41 (51.9)	35 (45.5)	34 (41.5)
	p30^k^	87 (13.7)	12 (14.8)	7 (9.1)	9 (12.3)	14 (17.3)	11 (12.8)	11 (13.9)	12 (15.6)	11 (13.4)
**Cigars**										
	Ever	206 (32.4)	27 (33.3)	29 (37.7)	29 (39.7)	22 (27.2)	20 (23.3)	28 (35.4)	25 (32.5)	26 (31.7)
	p30	61 (9.6)	2 (7.4)	11 (14.3)	4 (5.5)	9 (11.1)	8 (9.3)	10 (12.7)	4 (5.2)	9 (11.0)
**Smokeless**										
	Ever	33 (5.2)	2 (2.5)	1 (1.3)	6 (8.2)	3 (3.7)	2 (2.3)	9 (11.4)	6 (7.8)	4 (4.9)
	p30	5 (0.8)	1 (1.2)	1 (1.3)	1 (1.4)	0 (0.0)	0 (0.0)	2 (2.5)	0 (0.0)	0 (0.0)
**Hookah**										
	Ever	354 (55.7)	42 (51.9)	43 (55.8)	34 (46.6)	46 (56.8)	52 (60.5)	46 (58.2)	44 (57.1)	47 (57.3)
	p30	116 (18.2)	16 (19.8)	15 (19.5)	8 (11.0)	15 (18.5)	18 (20.9)	15 (19.0)	15 (19.5)	14 (17.1)
**e-Cigarettes**										
	Ever	171 (26.9)	22 (27.2)	19 (24.7)	19 (26.0)	21 (25.9)	26 (30.2)	22 (27.9)	20 (26.0)	22 (26.8)
	p30	50 (7.9)	5 (6.2)	5 (6.5)	7 (9.6)	7 (8.6)	13 (15.1)	5 (6.3)	6 (7.8)	2 (2.4)
Marijuana		161 (25.3)	21 (25.9)	24 (31.2)	15 (20.6)	21 (25.9)	22 (25.6)	24 (30.4)	17 (22.1)	17 (20.7)
Poly-tobacco use^k^		300 (47.2)	43 (53.1)	34 (44.2)	35 (48.0)	39 (48.2)	40 (46.5)	42 (53.2)	34 (44.2)	33 (40.2)
**Susceptibility to use^l^**										
	Cigarettes	46 (13.2)	7 (15.9)	2 (4.7)	2 (4.4)	9 (19.6)	7 (18.3)	6 (15.8)	4 (9.5)	9 (18.8)
	Cigars	96 (22.7)	14 (24.1)	4 (7.8)	7 (14.9)	19 (29.2)	18 (26.1)	10 (16.7)	10 (18.2)	14 (23.7)
	Smokeless	80 (13.3)	14 (17.7)	5 (6.6)	7 (10.5)	13 (16.7)	10 (11.9)	8 (11.4)	10 (14.1)	13 (16.7)
	Hookah	85 (30.1)	19 (48.7)	6 (17.7)	12 (30.8)	10 (28.6)	11 (32.4)	8 (24.2)	6 (18.2)	13 (37.1)
	e-Cigarettes	125 (26.9)	21 (35.59)	13 (22.4)	11 (20.4)	16 (26.7)	13 (21.7)	18 (31.6)	14 (24.6)	19 (31.7)
Use marijuana and tobacco		275 (43.2)	40 (49.4)	39 (50.6)	26 (35.6)	34 (42)	38 (43.7)	32 (40.5)	31 (40.3)	35 (42.7)
Secondhand smoke in house		68 (10.7)	10 (12.3)	9 (11.7)	10 (13.7)	7 (8.6)	6 (6.9)	4 (5.1)	12 (15.6)	10 (12.2)
Have friends who use tobacco		566 (89.0)	76 (93.8)	67 (87.0)	60 (82.2)	68 (84.0)	80 (92.0)	69 (87.3)	72 (93.5)	74 (90.2)

^a^Results that include *Ever* product use followed by *Past 30 days* use (p30).

^b^Randomization of participants to 8 groups of short message service text messages.

^c^CGE: complex, gain-framed, emotional.

^d^CGR: complex, gain-framed, rational.

^e^CLE: complex, loss-framed, emotional.

^f^CLR: complex, loss-framed, rational.

^g^SGE: simple, gain-framed, emotional.

^h^SGR: simple, gain-framed, rational.

^i^SLE: simple, loss-framed, emotional.

^j^SLR: simple, loss-framed, rational.

^k^Refers to the concurrent use of multiple tobacco products among participants at any time.

^l^Susceptibility to use is measured with nonusers only.

**Table 3:** Week 1 manipulation check outcomes for the total sample and by treatment arm.

Outcomes^a,b^	Mean (SD)									*P* value^k^
	Total	CGE^c^	CGR^d^	CLE^e^	CLR^f^	SGE^g^	SGR^h^	SLE^i^	SLR^j^	
Perceived message-framing as a loss (8-point scale)	5.20 (3.22)	3.74 (2.23)	4.39 (3.00)	5.74 (3.25)	7.58 (2.86)	4.07 (2.66)	4.17 (3.04)	6.06 (3.33)	6.00 (3.41)	<.001
Perceived complexity level (8-point scale)	2.98 (2.02)	3.07 (2.12)	2.81 (2.26)	3.34 (2.33)	3.60 (2.14)	2.45 (1.54)	2.86 (1.95)	3.05 (1.96)	2.78 (1.81)	.05
Perceived emotional level (8-point scale)	3.35 (2.23)	3.72 (2.13)	2.24 (1.52)	4.39 (2.72)	3.39 (2.33)	3.62 (2.22)	2.80 (1.90)	3.82 (2.28)	2.86 (1.97)	<.001
Perceived credibility (8-point scale)	7.57 (2.01)	7.43 (1.85)	8.01 (1.78)	7.60 (1.90)	7.87 (1.80)	6.98 (2.45)	7.70 (1.96)	7.36 (2.01)	7.64 (2.09)	.10
Enjoyment of messages (8-point scale)	5.90 (1.49)	5.87 (1.46)	5.78 (1.37)	6.13 (1.57)	5.93 (1.46)	5.93 (1.54)	5.78 (1.47)	5.96 (1.56)	5.80 (1.53)	.90
Perceived relevance (5-point scale)	2.37 (0.85)	2.37 (0.87)	2.40 (0.91)	2.38 (0.81)	2.57 (0.79)	2.22 (0.81)	2.41 (0.83)	2.26 (0.94)	2.40 (0.81)	.40
Perceived readability (5-point scale)	3.46 (0.78)	3.41 (0.81)	3.44 (0.84)	3.51 (0.75)	3.45 (0.85)	3.53 (0.70)	3.50 (0.75)	3.36 (0.84)	3.49 (0.71)	.93

^a^Missing values are not presented in this table. Out of 636 participants, 530 (530/636, 83.3%) completed the manipulation check survey.

^b^Participants were randomized to one of the 8 treatment arms, describing the type of messages.

^c^CGE: complex, gain-framed, emotional.

^d^CGR: complex, gain-framed, rational.

^e^CLE: complex, loss-framed, emotional.

^f^CLR: complex, loss-framed, rational.

^g^SGE: simple, gain-framed, emotional.

^h^SGR: simple, gain-framed, rational.

^i^SLE: simple, loss-framed, emotional.

^j^SLR: simple, loss-framed, rational.

^k^Significance testing with analysis of variance.

**Table 4:** Baseline risk communication outcomes for the entire sample and by treatment arm.

Outcome^a^	Mean (SD)								
	Total	CGE^b^	CGR^c^	CLE^d^	CLR^e^	SGE^f^	SGR^g^	SLE^h^	SLR^i^
Perceived risk of using cigarettes	3.69 (0.55)	3.76 (0.49)	3.68 (0.52)	3.78 (0.35)	3.68 (0.6)	3.57 (0.65)	3.70 (0.56)	3.75 (0.47)	3.60 (0.63)
Perceived risk of using cigars	3.61 (0.56)	3.67 (0.49)	3.64 (0.5)	3.63 (0.52)	3.62 (0.56)	3.51 (0.62)	3.65 (0.56)	3.62 (0.54)	3.52 (0.67)
Perceived risk of using smokeless tobacco	3.48 (0.60)	3.49 (0.57)	3.44 (0.60)	3.59 (0.49)	3.47 (0.68)	3.45 (0.61)	3.48 (0.60)	3.56 (0.53)	3.38 (0.68)
Perceived risk of using hookah	3.09 (0.8)	3.00 (0.87)	3.15 (0.71)	3.37 (0.72)	3.09 (0.79)	2.99 (0.84)	3.13 (0.79)	3.11 (0.77)	3.01 (0.85)
Perceived risk of using e-cigarettes	3.06 (0.83)	2.98 (0.82)	3.17 (0.65)	3.31 (0.77)	3.08 (0.8)	2.98 (0.9)	3.11 (0.84)	3.04 (0.84)	2.96 (0.94)
Perceived personal benefits of e-cigarettes	0.88 (0.76)	0.87 (0.77)	0.88 (0.73)	1.02 (0.87)	0.76 (0.65)	0.91 (0.8)	0.85 (0.77)	0.89 (0.72)	0.85 (0.74)
Perceived general benefits of e-cigarettes	1.41 (0.69)	1.47 (0.51)	1.53 (0.68)	1.42 (0.69)	1.39 (0.72)	1.45 (0.78)	1.31 (0.77)	1.37 (0.73)	1.38 (0.64)
Perceived addictiveness of products	1.23 (0.59)	1.23 (0.53)	1.26 (0.56)	1.33 (0.59)	1.21 (0.64)	1.24 (0.55)	1.18 (0.62)	1.23 (0.61)	1.18 (0.61)
Perceived popularity of tobacco use	2.44 (1.13)	2.57 (1.11)	2.5 (1.05)	2.33 (1.21)	2.36 (1.15)	2.47 (1.09)	2.49 (1.06)	2.28 (1.19)	2.48 (1.2)

^a^Participants were randomized to one of the 8 treatment arms, describing the type of messages.

^b^CGE: complex, gain-framed, emotional.

^c^CGR: complex, gain-framed, rational.

^d^CLE: complex, loss-framed, emotional.

^e^CLR: complex, loss-framed, rational.

^f^SGE: simple, gain-framed, emotional.

^g^SGR: simple, gain-framed, rational.

^h^SLE: simple, loss-framed, emotional.

^i^SLR: simple, loss-framed, rational.

## Discussion

### Overview

The Project Debunk trial will evaluate a comprehensive campaign delivered by mobile phone for increasing tobacco risk perception among a large sample of young adults in community college, including both tobacco users and nonusers. In particular, the trial will identify which structures of SMS text messages, if any, have the strongest effect on increasing the perceived risk of using conventional tobacco products and NETP. The results of this study will form the basis of an evidence-based resource that future researchers and practitioners could modify for use among their populations of interest.

To the best of our knowledge, this is the first published mHealth protocol for a trial that assesses the effect of a comprehensive and evidence-based mobile phone text messaging campaign for tobacco risk communication. This protocol summarizes the design and describes the planned evaluation of Project Debunk. Going beyond a simple presentation of our future study procedures, the protocol also presents the results from our baseline data. In particular, baseline information confirms that a substantial proportion of young adults at community colleges continue to smoke cigarettes, in addition to using NETP such as e-cigarettes and hookah. There were no differences among the treatment arms with respect to sociodemographic or tobacco-related characteristics. In addition, the treatment arms did not differ at the baseline with respect to the perceived risk of using any tobacco product. Preliminary results also show that we have successfully manipulated the 8-message structure combinations with our study sample. This is evident from treatment arm differences with respect to perceived message loss framing, emotional level, and complexity. All 8-message structure combinations were found to be enjoyable, easy to read, and credible.

### Anticipated Results

On the basis of previous pilot data collected by our team [62], we anticipate adequate feasibility and satisfaction among participants. In a previous study that we conducted with young adult college students [61], the recruitment rate was high (80.1%) and participants reported positive changes in their perceived risk of tobacco use. We anticipate similar results in Project Debunk for all groups. We project that all message structure combinations will result in an increase in perceived risk of using tobacco products. As suggested by recent reports [4,87], we expect higher levels of perceived risk of using combustible cigarettes compared with NETP such as e-cigarettes and hookah. In addition, change over time in perceived risk is expected to be lower for combustible cigarettes, compared with e-cigarettes and hookah. We cannot predict or anticipate specific results with respect to which message structure is most effective in improving tobacco risk perception. This study will be the first to provide empirical evidence that highlights the importance of one message structure over another in the context of tobacco risk communication. Once the successful types of text messages have been identified, our future plan is to introduce the messages in the context of an advanced digital intervention that can effectively communicate tobacco risk.

### Strengths and Limitations

We will address the anticipated difficulties described in previous studies of mobile phone text messaging in young-adult populations [88-90], such as participant retention, in several ways: regular communication with participants and continuous reminders via phone, and compensation (gift cards) at project completion. This study has a convenience sample. Nevertheless, our sample is representative of the community college population in age, gender, and ethnicity. It also involves a heterogeneous ethnic distribution, with a proportion of tobacco users and demographics that are similar to that of young adults in the state of Texas [91].

### Conclusions

It is evident that young adult tobacco users and nonusers are interested in mHealth programs that help them learn about tobacco risks [62]. Moreover, as a mass media strategy, mHealth programs offer the potential to greatly increase the reach of young adults. If our results suggest that a specific mobile phone text message structure is most effective for helping young adults accurately perceive tobacco risk, this would provide evidence to include such text messages as part of larger technology-based campaigns such as smartphone apps, entertainment-based campaigns, and social media. These findings would also provide a deeper understanding of the factors that drive change in the perceived risk of using tobacco and improve the design of our text messages. Considering the wide variety of tobacco products studied in the trial, the results will highlight any potential differences between the products. With the use of mHealth text messaging, the results of this study will reveal the best strategies to efficiently and widely communicate risk to young adults and ultimately prevent tobacco use in this age demographic.

## Appendix

Multimedia Appendix 1 Peer-review report from the Food and Drug Administration of the National Institutes of Health.

Multimedia Appendix 2 Examples of text messages.

Multimedia Appendix 3 Checklist for Reporting Results of Internet E-Surveys (CHERRIES).

Multimedia Appendix 4 Description of the main measures.

## Abbreviations

AIM: assessment, intervention, and measurement
ANOVA: analysis of variance
CGE: complex, gain-framed, emotional
CGR: complex, gain-framed, rational
CLE: complex, loss-framed, emotional
CLR: complex, loss-framed, rational
e-cigarettes: electronic cigarettes
ELM: elaboration likelihood model
HCC: Houston Community College
mHealth: mobile health
NETP: new and emerging tobacco products
PC1: postcampaign 1
PC2: postcampaign 2
SGE: simple, gain-framed, emotional
SGR: simple, gain-framed, rational
SLE: simple, loss-framed, emotional
SLR: simple, loss-framed, rational
SMS: short message service
TX TCORS: Tobacco Center of Regulatory Science on Youth and Young Adults
