# Supplementary material for: Mobile Phone Text Messaging for Tobacco Risk Communication Among Young Adult Community College Students: Protocol and Baseline Overview for a Randomized Controlled Trial
Source: JMIR Res Protoc. 2018 Oct 15;7(10):e10977. doi: 10.2196/10977 (PMC6231779; doi:10.2196/10977)
Supplement: Multimedia Appendix 1 [file resprot_v7i10e10977_app1.pdf]

SUMMARY STATEMENT *Release Date:* 05/25/2013

Application Number: 1 P50 CA180906-01
Formerly: 1P50DA036116-01

Principal Investigator
PERRY, CHERYL L PHD
Applicant Organization: UNIVERSITY OF TEXAS HLTH SCI CTR HOUSTON
Review Group:
Center for Scientific Review Special Emphasis Panel
RFA DA13-003: Tobacco Centers of Regulatory Science for Research Relevant to the Family Smoking Prevention and Tobacco Control Act (P50)
Meeting Date: 05/09/2013
Requested Start: 09/01/2013

Project Title: Tobacco Center of Regulatory Science on Youth and Young Adults

Next Steps: Visit http://grants.nih.gov/grants/next_steps.htm
Human Subjects: 30-Human subjects involved - Certified, no SRG concerns
Animal Subjects: 10-No live vertebrate animals involved for competing appl.
Gender: 1A-Both genders, scientifically acceptable
Minority: 1A-Minorities and non-minorities, scientifically acceptable
Children: 1A-Both Children and Adults, scientifically acceptable
Clinical Research - not NIH-defined Phase III Trial

RESUME AND SUMMARY OF DISCUSSION: This application proposes to examine tobacco use by youth and young adults and the marketing of tobacco products to them. This will be accomplished in three research projects.

PROJECT 3: Informing and Correcting Perceptions Regarding Tobacco Products in Young Adults

PROJECT LEADER: Alexander V. Prokhorov

Project 3 will examine the attitudes of young adults about conventional, new and emerging tobacco products. It will also examine the efficacy of using different types of SMS text messages about tobacco products in providing information to young adults about the risks of tobacco use. If it is efficacious, SMS text messages could be used by the FDA to deliver information about the risks associated with tobacco use to youth.

CRITIQUE 1:
Overall Impact: The proposed study will investigate how text messages, which communicate the dangers of tobacco (both cigarettes and alternative products), might influence the perception of tobacco‘s harm among young adults. This study will document existing attitudes and awareness of tobacco harms among a community college population and then experiment with various message characteristics and examine moderators of message efficacy. Successful completion of the aims will document current awareness and perception of tobacco‘s harms and potentially illustrate characteristics of messages that can modify these perceptions. Documenting the baseline awareness of tobacco‘s harms has relevance to the FDA‘s mission under the FSTCA, as does increasing the understanding of effective messaging as it could guide FDA communications. Several weaknesses tempered enthusiasm for the proposed work. First, it is unclear how a text message intervention aimed at a general population of young adults could be used effectively in the real world beyond the study by the FDA or even by other parties. Second, the first aim seems as it could be more effectively achieved via this Center proposal‘s second project, a much larger scale survey of young adults which includes community college campuses. Third it is not clear why the study population is limited to community college students and further those attending two community colleges near the investigator‘s institutions. Since the intervention is electronic it could be tested in a more broad, diverse young adult population with relative ease which would enhance generalizability.

1. Significance:

Strengths

Assessing awareness of dangers and attitudes toward tobacco products, especially emerging ones, among young adults would be useful information. As would assessing moderators of these attitudes. This could enable FDA to craft messages and regulate how products are advertised.
1 P50 CA180906-01 39 ZRG1 BDCN-A (40) PERRY, C

Developing effective messages than can communicate the dangers of tobacco products and alter attitudes would be a significant outcome that would inform regulatory science.

Weaknesses
While developing effective message tones, styles, and characteristics would be significant and useful information for the FDA to deliver information via various modes, and while testing messages via texts can be an effective, cheap, and quick way to do this, the application does not describe how text messages might actually be used in the real world by the FDA and it is hard to imagine how this might work. So even if text message are effective it is unclear how this could actually be implemented or would be relevant for FDA use or FDA regulation. Would the FDA be sending texts to random young adults? Even outside of the FDA, how could it be implemented -- would community colleges get the cell phone numbers of all students or students who were at risk and text them? Those two options would be annoying to recipients and costly outside of this study where participants get reimbursed for cell expenses (since texts typically cost the receiver). Perhaps people could sign up to get tobacco education texts, but who would do that?

2. Investigator(s):

Strengths
Dr. Pokhorov has experience in tobacco interventions for young adults as well developing tailored electronic messages for health promotion.

Other members of the team have strengths in communication and electronic messaging and well as health behavior theory.

Weaknesses
In the budget, page 235, of this application Drs. Vidrine and Calabro are listed as “Co-PI,” Dr. Prokhorov is listed as “PD/PI.” It appears that this is an error and that Drs. Vidrine and Calabro are actually Co-Is as they are listed in the budget justification (page 238). Otherwise a multiple PI plan is missing and needed.

Investigators seem to have experience in the areas of tobacco, young adult research, communication research and electronic communication however it is a bit of a leap to assume this as their preliminary studies subsection gave very little detail or concrete examples of their previous work.

3. Innovation:

Strengths
A better understanding of young adult perceptions of tobacco harm would aid in regulating how products are advertised.

This proposal would advance the science of what messaging content and characteristics might stick with young adults.

Weaknesses
There is an assumption that text messages would be an appropriate mode for the FDA to use in communication but there is no support for that.

There is no attempt to discover what mode of communication (text or others) might be most appropriate on both ends of the communication – both for the FDA to use to broadcast messages and for young adults and how they might prefer receiving messages.

4. Approach:
Strengths

Survey measures well-detailed, appropriate, and concise. Conducting the survey via cell phone is a strength given that participants will be interacting with the study intervention via cell phone. This survey mode should promote a good response rate. The focus groups to develop messages is a strength. The factorial design is an efficient way to test multiple message characteristics.

Weaknesses
What if all of the students who volunteer for the study already perceive the tobacco products to be risky? How can change in perceptions be measured? As the application mentioned, over ¾ of high school seniors in Monitoring the Future perceive "great risk" in smoking a pack of cigarettes a day. There might not be room for change.

Given the recruitment plan, the sample may be very nuanced and the generalizability of Aim 1's results could be limited.

It is unclear why the hypothesis behind Aim 1 is related to comparing risk perception of emerging products to the risk perception of conventional products. That comes with the assumption that the risk perception of conventional products is adequately high. Instead it would be important to document the risk perception of various products, all of which are risky and should be perceived as such.

Concerns about adequate sample size to achieve Aim 3. The "rule of thumb" of 10:1 events to variables is not a rigorous standard and it is unclear what detectable difference it assumes.

Concerns that with the cross-over design, the three-month washout period is inadequate. It is unknown how long these messages "last." One would hope that they would last longer than three months.

Application makes several analogies to the effect the Fairness Doctrine had on perception of the dangers of smoking. Given this, would it not make more sense to use the same channel/s for message delivery that the tobacco companies are using rather than texting?

5. Environment:
Strengths
The environment appears to be very supportive for this type of research.

Weaknesses
None noted.

Protections for Human Subjects:
Acceptable Risks and/or Adequate Protections
Focus group and pretest participants should be included in Human Subjects section.

Data and Safety Monitoring Plan (Applicable for Clinical Trials Only):
Not Applicable (No Clinical Trials)

Inclusion of Women, Minorities and Children:
G1A - Both Genders, Acceptable
M1A - Minority and Non-minority, Acceptable
C1A - Children and Adults, Acceptable

Vertebrate Animals:
Not Applicable (No Vertebrate Animals)

Biohazards: Not Applicable (No Biohazards)
Applications from Foreign Organizations: Not Applicable (No Foreign Organizations)
Select Agents: Not Applicable (No Select Agents)
Resource Sharing Plans: Not Applicable (No Relevant Resources)
Budget and Period of Support: Recommended budget modifications or possible overlap identified:
Budget appears high for scope of work, as does the time. Recommend a cutback on investigator effort and three or four year timeline.

CRITIQUE 2:

Overall Impact: It is highly likely that this project will exert an influence on the regulation of tobacco products and protection of public health. The project will provide a critical new understanding of conventional and new/emerging tobacco knowledge and usage among young adults. The project will achieve this by focusing on highly susceptible community college students and providing valuable insight into how to calibrate and compose highly accurate text messages to counter misleading tobacco marketing, educate young adults about tobacco use, and better convey the risks associated with new/emerging tobacco products. This project has a high impact potential given that it uses text messaging to deliver health messages to a very hard-to-reach segment of the population. It will make an impact by providing the FDA with a tested library of potentially scalable text messages that can potentially be used with other groups of young adults throughout the country. In addition, the project's statistical analysis will provide valuable new data pertaining to awareness, attitudes, perceptions, and receptivity of the harmful health ramifications of conventional and new/emerging tobacco products. Given the considerable strengths of the project, the only potential weakness concerns whether the project's five-year timeline could potentially be sped up given the urgency of the research need and the project organization's institutional capacity and proven experience.

1. Significance:
Strengths
The project addresses a range of research priorities including better understanding the diversity of tobacco products, behaviors of individuals using new/emerging tobacco products, and a more sophisticated understanding of tobacco products public health communication.

The project addresses significant information gaps pertaining to the use of mobile phone messaging to inform young adults about the risk of conventional cigarettes and new/emerging tobacco.

Weaknesses
A portion of the project is aimed at better understanding young adult usage of new/emerging tobacco products though only a relative small portion (18%) of young adults actually report using these products.

2. Investigator(s):
Strengths
The PI, Co-Investigators, and other researchers are all highly suited to the project. Each brings a unique background and experience that complements the aims of the project.

Investigators have extensive collective experience designing text messages and recruiting and retaining college students in a text-message-based program.

The PI has extensive experience conducting tobacco-related studies, particularly examining usage among high-school, community college, and university students.

The PI brings highly complementary experience given involvement with Project ACTION which uses cell phone-delivered text messages as part of a smoking cessation program and extensive studies of risk perceptions of new/emerging tobacco among youth.

Co-Investigators have highly complementary and integrated experience.

Weaknesses None noted.

3. Innovation:
Strengths
The project seeks to shift current research by utilizing technology to fully examine an overlooked and highly-vulnerable segment of the population.

The project utilizes a novel, inexpensive, and highly-effective means of communication and data-gathering (text-messaging via mobile phones).

The project is scalable to other susceptible groups of young adults across the United States.

4. Approach:

Strengths
The project's overall strategy, methodology, and analyses are very well-reasoned and highly suited to the specific aims of the project.

The project draws upon and is a logical advancement of previous and on-going work including Project ASPIRE, Project "Look At Your Health," and Project ACTION.

The project team has significant experience working with young adults, designing text messages, and completing studies using cell phones.

Excellent use of prior experience to keep surveys short, easily accessible (via cell phone), and ultimately more effective.

Weaknesses
A separate text messaging investigation noted that young adults reported "high satisfaction with five messages per day." This seems rather high in terms of the likelihood of young adults actually taking the time to read and absorb text message content. This project, however, recommends a dosage of two texts per day. This is clearly lower yet some skepticism remains that young adults will remain actively engaged with this daily blitz of information.

5. Environment:

Strengths
The scientific environment in which the work will be done is well suited for this project and is highly likely to contribute to the project's probability of success.

The institutional support and physical resources necessary are readily available and more than adequate for this project.

It is highly likely that the project will benefit from the scientific environment, unique subject populations, and collaborative arrangements.

Weaknesses None noted.

Protections for Human Subjects:
Acceptable Risks and/or Adequate Protections

Excellent consideration of, and experience with, recruitment, informed consent, and protections against risk.

Data and Safety Monitoring Plan (Applicable for Clinical Trials Only): Acceptable

Data protection sufficiently reviewed on behalf of both participants and overall project management.

Inclusion of Women, Minorities and Children:
G1A - Both Genders, Acceptable
M1A - Minority and Non-minority, Acceptable
C1A - Children and Adults, Acceptable
Vertebrate Animals:
Not Applicable (No Vertebrate Animals)
Biohazards: Not Applicable (No Biohazards)
Applications from Foreign Organizations: Not Applicable (No Foreign Organizations)
Select Agents: Not Applicable (No Select Agents)
Resource Sharing Plans: Acceptable Data Sharing Plan fulfills the aims of the project and will likely benefit the overall research community.
Budget and Period of Support: Recommend as Requested

CRITIQUE 3:

Overall Impact: The proposed study addresses an important problem area. The Elaboration Likelihood Model of Persuasion will be used to guide messages delivered via mobile phones to youths at risk for tobacco use to establish the effectiveness of alternate message frames. The guiding theoretical framework is innovative and there is a strong need for development of effective interventions of this nature. The team is strong and relevant prior experience. However, overall enthusiasm is tempered by a lack of developmental research which establishes the potential of the approach.

1. Significance:

Strengths
The proposed project addresses an important problem area; the vulnerability of youth to tobacco marketing.

If successful, the study will identify an effective means of delivering tobacco prevention messages to young adults via text messaging.

Weaknesses
The text messaging intervention will be guided by a potentially valuable theoretic approach. However, no empiric support is provided which establishes its potential as applied in the proposed study.

2. Investigator(s):
Strengths
The investigative team is strong, with extensive pertinent experience.

Weaknesses
While the team has extensive experience in the area, results from the prior studies do not inform the intervention that will be tested in the proposed study.

3. Innovation:
Strengths
The guiding theoretical framework is innovative as is the use of theory to guide text message interventions. Regrettably, few text messaging interventions that have been tested to date are theoretically grounded.

Weaknesses
None noted.

4. Approach:
Strengths
The approach is generally well developed and suitable for achieving the aims of the study.

Weaknesses
While the guiding theory is interesting and logically congruent, the large scale study seems premature without prior developmental testing to demonstrate its promise as applied to tobacco control messages targeting youth. The theory guides framing of messages, but no evidence or theoretic justification is provided for the pattern of delivery proposed.

5. Environment:
Strengths
Strong.

Weaknesses
None noted.

Protections for Human Subjects:
Acceptable Risks and/or Adequate Protections 1 P50 CA180906-01 46 ZRG1 BDCN-A (40) PERRY, C

Data and Safety Monitoring Plan (Applicable for Clinical Trials Only):

Not Applicable (No Clinical Trials)
Inclusion of Women, Minorities and Children:
G1A - Both Genders, Acceptable
M1A - Minority and Non-minority, Acceptable
C1A - Children and Adults, Acceptable
Vertebrate Animals:
Not Applicable (No Vertebrate Animals)
Biohazards:
Not Applicable (No Biohazards)
Applications from Foreign Organizations:
Not Applicable (No Foreign Organizations)
Select Agents:
Not Applicable (No Select Agents)
Resource Sharing Plans:
Not Applicable (No Relevant Resources)
Budget and Period of Support:
Recommend as Requested

NIH has modified its policy regarding the receipt of resubmissions (amended applications). See Guide Notice NOT-OD-10-080 at http://grants.nih.gov/grants/guide/notice-files/NOT-OD-10-080.html. The impact/priority score is calculated after discussion of an application by averaging the overall scores (1-9) given by all voting reviewers on the committee and multiplying by 10. The criterion scores are submitted prior to the meeting by the individual reviewers assigned to an application, and are not discussed specifically at the review meeting or calculated into the overall impact score. Some applications also receive a percentile ranking. For details on the review process, see http://grants.nih.gov/grants/peer_review_process.htm#scoring.
