# Supplementary material for: Mobile Phone Text Messaging for Tobacco Risk Communication Among Young Adult Community College Students: Protocol and Baseline Overview for a Randomized Controlled Trial
Source: JMIR Res Protoc. 2018 Oct 15;7(10):e10977. doi: 10.2196/10977 (PMC6231779; doi:10.2196/10977)
Supplement: Multimedia Appendix 2 [file resprot_v7i10e10977_app2.pdf]

Multimedia Appendix 2:

Examples of text messages used in our protocol.

	Gain-framed		Loss-framed	
	**Simple**	**Complex**	**Simple**	**Complex**
**Emotional**	Yummy, pie! Nonsmokers can appreciate every single bite of homemade apple pie since the nicotine in cigs hasn’t messed up their taste buds! :P	Be astute! Savvy individuals who do not smoke hookah prevent exposure to disgusting tar and carcinogens that the gullible ASSUME are filtered through the pipe’s water. :)	Mike had a hot date on Friday but wouldn’t stop smoking cigs with his pals. Now he has rotting yellow teeth & an imaginary date. :(	Hookah smokers are more susceptible to contracting unsightly oral herpes than nonsmokers since mouthpieces are ~ carriers for everyone's disgusting pathogens. :(
**Rational**	With 7000+ toxic chemicals in cig smoke, the chances of a nonsmoker getting cancer is really low. Why? They aren’t exposed to 60+ cancer-causing chemicals.	Individuals who choose not to smoke hookah prevent exposure to tar containing carcinogens. Despite popular belief, ~ these harmful substances are not filtered through water.	Hookah users expose their bodies to a lot of smoke since hookah creates 200x more smoke than a cig.	Why do the 60+ carcinogens in cigarettes pose a direct threat to smokers? ~ Because smokers inhale those carcinogens, raising their chances of developing cancer.
