# Supplementary material for: Mobile Phone Text Messaging for Tobacco Risk Communication Among Young Adult Community College Students: Protocol and Baseline Overview for a Randomized Controlled Trial
Source: JMIR Res Protoc. 2018 Oct 15;7(10):e10977. doi: 10.2196/10977 (PMC6231779; doi:10.2196/10977)
Supplement: Multimedia Appendix 3 [file resprot_v7i10e10977_app3.pdf]

Multimedia Appendix 3:

Checklist for Reporting Results of Internet E-Surveys (CHERRIES) Design

The e-surveys of the present study were aimed to collect data from young adults attending community college (ages 18-25 years). For the purpose of the present protocol, the surveys were administered at baseline and at one week during campaign exposure. This checklist will be reported one more time, once the study is completed with the main outcomes of the trial.

Development and Pre-testing:

All the surveys were designed using Qualtrics [1]. The baseline and week-1 manipulation check measures were chosen based on previous research in media communication, human-computer communication, and public health [2-18]. The surveys were pre-tested for usability and technical functionality during a pilot qualitative study (focus group discussions). During the pilot study, participants were encouraged to ask questions concerning the surveys and report any concerns to the research team [19].

IRB Approval, Informed Consent Process, Recruitment, and Survey Administration

The study involving these surveys has been approved by the institutional review board. The initial contact with potential participants was made face-to-face on campus. We set up recruitment stations or booths equipped with a highly visible logo of the research institution. Printed materials (e.g., posters and fliers) announcing the study were displayed in common areas, such as student lounges. During participant recruitment at each campus, the research staff explained the purpose of the study to students and answered their questions. Our announcements explained the purpose of the study, the length of the study, method of accessing the study, risks/benefits of the study, and statement of voluntary participation. We have obtained consent from young adults before their participation. The consent forms are retained by the research team and secured in a locked file cabinet. Participants are given copies of the consent forms. Following consent, participants completed the baseline survey on their personal phones. After one week of receiving text messages from the campaign, participants completed the week-1 manipulation check survey. The surveys did not allow for the participants to review answers. Participants could not go to a previous page to change their answers.

Response Rate:

Considering the need for full privacy, IP addresses were not collected for participants. Instead, a unique participant was determined based on ID numbers assigned to them on their first day of participation. The participation rate for the baseline survey and the manipulation check survey was 100%. The completion rate for the baseline survey was 100%, and the manipulation check survey was 98.70%. Participants could not skip questions in the surveys. In order to receive compensation for a specific survey, participants had to complete the survey.

Preventing Multiple Entries:

While the surveys were closed, they did involve a login to prevent duplicate entries. For all surveys, IP addresses were not collected based on the requirements of the institutional review board. However, duplicate database entries having the same ID number were eliminated before analysis, such that the first entry was kept and the second entry was eliminated.

Analysis:

All the data, including incomplete questionnaires, were analyzed. All participants provided answers until the last page of the surveys. No statistical method was needed to correct for missing values.

References:

1. Qualtrics Labs. Qualtrics Survey Software: Handbook for Research Professionals 2012. Qualtrics Labs, Incorporated, 2012 ISBN: 0984932801.
2. Wray RJ, Jupka K, Berman S, Zellin S, Vijaykumar S. Young adults' perceptions about established and emerging tobacco products: results from eight focus groups. Nicotine Tob Res. 2012 Feb;14(2):184-90. PMID: 22110049.
3. Rumpf H-J, Meyer C, Hapke U, John U. Screening for mental health: validity of the MHI-5 using DSM-IV Axis I psychiatric disorders as gold standard. Psychiatry Res. 2001;105(3):243-53. PMID: 11814543
4. Johnston LD, O'Malley PM, Bachman JG, Schulenberg JE, Miech RA. Monitoring the Future national survey results on drug use, 1975-2015: Volume II, college students and adults ages 19-55. 2016. Bethesda, Md: National Institute on Drug Abuse.
5. Kebede M, Zeleke A, Asemahagn M, Fritz F. Willingness to receive text message medication reminders among patients on antiretroviral treatment in North West Ethiopia: A cross-sectional study. BMC Med Inform Decis Mak. 2015;15(1):65. PMID: 26268394
6. Kahlor L, Dunwoody S, Griffin RJ, Neuwirth K. Seeking and processing information about impersonal risk. Sci Commun. 2006;28(2):163-94. Doi: 10.1177/1075547006293916
7. Mason M, Cheung I, Walker L. Substance use, social networks, and the geography of urban adolescents. Subst Use Misuse. 2004;39(10-12):1751-77. PMID: 15587950
8. Berwick DM, Murphy JM, Goldman PA, Ware Jr JE, Barsky AJ, Weinstein MC. Performance of a five-item mental health screening test. Med Care. 1991;29(2):169-76. PMID: 1994148
9. Fellner B, Holler M, Kirchler E, Schabmann A. Regulatory focus scale (RFS): Development of a scale to record dispositional regulatory focus. Swiss J Psychol. 2007;66(2):109-16. Doi: 10.1024/1421-0185.66.2.109
10. Stephenson MT, Hoyle RH, Palmgreen P, Slater MD. Brief measures of sensation seeking for screening and large-scale surveys. Drug Alcohol Depend. 2003;72(3):279-86. PMID: 14643945
11. Nelson W, Reyna VF, Fagerlin A, Lipkus I, Peters E. Clinical implications of numeracy: theory and practice. Ann Behav Med. 2008;35(3):261-74. PMID: 18677452
12. Toll BA, O'malley SS, Katulak NA, Wu R, Dubin JA, Latimer A, et al. Comparing gain- and loss-framed messages for smoking cessation with sustained-release bupropion: a randomized controlled trial. Psychol Addict Behav. 2007;21(4):534. PMID: 18072836
13. See YHM, Petty RE, Evans LM. The impact of perceived message complexity and need for cognition on information processing and attitudes. J Res Pers. 2009;43(5):880-9. Doi: 10.1016/j.jrp.2009.04.006
14. Vidrine JI, Simmons VN, Brandon TH. Construction of smoking-relevant risk perceptions among college students: The influence of need for cognition and message content. J Appl Soc Psychol. 2007 Jan;37(1):91-114. Doi: 10.1111/j.0021-9029.2007.00149.x.
15. Eastin MS. Credibility assessments of online health information: The effects of source expertise and knowledge of content. J Comput-Mediat Comm. 2001;6(4):0-0. Doi: 10.1111/j.1083-6101.2001.tb00126.x
16. Cyr D, Head M, Ivanov A. Perceived interactivity leading to e-loyalty: Development of a model for cognitive–affective user responses. Int J Hum-Comput Int St. 2009;67(10):850-69. Doi: 10.1016/j.ijhcs.2009.07.004
17. Nysveen H, Pedersen PE, Thorbjørnsen H. Intentions to use mobile services: Antecedents and cross-service comparisons. J Acad Market Science. 2005;33(3):330-46. Doi: 10.1177/0092070305
18. Coursaris CK, Sung J. Antecedents and consequents of a mobile website's interactivity. New Media Soc. 2012;14(7):1128-46. Doi: 10.1177/1461444812439552

19. Prokhorov AV, Machado TC, Calabro KS, Vanderwater EA, Vidrine DJ, Pasch KP, et al. Developing mobile phone text messages for tobacco risk communication among college students: a mixed methods study. BMC Public Health. 2017;17(1):137. PMID: 28143432
