# Supplementary material for: Mobile Phone Text Messaging for Tobacco Risk Communication Among Young Adult Community College Students: Protocol and Baseline Overview for a Randomized Controlled Trial
Source: JMIR Res Protoc. 2018 Oct 15;7(10):e10977. doi: 10.2196/10977 (PMC6231779; doi:10.2196/10977)
Supplement: Multimedia Appendix 4 [file resprot_v7i10e10977_app4.pdf]

Multimedia Appendix 4:

Measures	Description and references	Cronbach's alpha
**Baseline**		
Economic status	A measure of one's comfort of living, with one question asking: "How would you describe your current financial situation?" with 4 answer choices spanning from "cannot meet basic expenses" to "live comfortably"	-
Numeracy ability	A measure of one's ability with understanding numbers in the context of medical statistics, such as chances and percentages [68].	-
Mental Health	This measure includes 5 items on psychological distress with the mental health inventory-5 (MHI-5) [60].	0.794
Receptivity to receiving text messages	Four dichotomous items assessing YA tendency to communicate via text messages [62].	-
Prevention focus level	This measure involves five items on the extent to which participants focus on the prevention of failure and safety [66].	0.425
Sensation seeking level	This is a trait indicating individuals' tendency to pursue sensory pleasure and excitement, by seeking novelty, complexity, and intense sensations. Sensation seekers love experience for its own sake, and may take risks in the pursuit of such experience [67].	0.706
Tendency for tobacco information seeking	This measure involves two items on participants' tendency to seek information about tobacco.	0.069[a]
**Weekly manipulation check**		
Perceived framing type of messages	With one item on an 8-point differential scale, participants are asked if they find the messages to be focused mostly on the benefits of not using tobacco or on the costs of using tobacco [69].	-
Perceived complexity level of messages	With one item on an 8-point differential scale, participants are asked if the messages seemed mostly simple or mostly complex [70].	-
Perceived emotional level of messages	With one item on an 8-point differential scale, participants are asked if the messages seemed mostly factual or mostly emotional [71].	-
Message credibility	With two items on an 8-point differential scale, participants are asked how credible and believable they find the messages [72].	0.597[a]
Message enjoyment	Six items on an 8-point differential scale, measuring how much participants find the messages to be entertaining, enjoyable, and fun [73, 74].	0.731
Perceived message relevance	Six items on a 5-point Likert scale from "not at all" to "very much", measuring how much participants find the messages relevant to them [75].	0.867
Perceived message readability	Designed by the research team with two items on a 5-point Likert scale from "not at all" to "very much", measuring how easy it is to understand the text messages (e.g., "the messages are hard to read").	0.374[a]
**7-day PC1 and 7-day PC2**		
Attention to the messages	Two items asking about participants' attention to the messages, with items such as "When I was reading the text messages, I paid attention to the messages more than to what was happening around me." Participants were asked if this was true for none of the messages, one to two messages, some of the messages, a lot of the messages, or all the messages [76].	-
Emotional involvement in message content	One items [76]: "I felt emotionally involved in the website." Answer choices were also ranging from "none of the messages" to "all of the messages".	-
Thought provocation	Four items asking how much participants were provoked to think of the content of the messages (e.g., "After reading the text messages, I	-

	found myself actively thinking about the content.") [76].	
Motivation to discuss the messages with others	Two items asking participants how motivated they are to talk to someone about the messages (e.g., "the text messages made me want to talk to someone about them.") [78, 79].	-
Discussions about the messages and tobacco with others	Participants were asked if they had did you have a conversation with someone about tobacco and the text messages during the past 30 days [79].	-
**Baseline, 3-month PC1 and 3-month PC2**		
Ever use of tobacco	For each tobacco product, participants were asked: "Have you ever tried [product name]?" Answers were yes or no.	-
Past 30-day use of tobacco	For each tobacco product, participants were asked: "During the past 30 days, on how many days did you use [product name]?" Answers were open-ended. This variable was dichotomized for the purpose of this protocol.	-
Perceived risk of using cigarettes	Each outcome had five items: the first item on a 4-point Likert scale, from no risk to great risk, asks respondents how much they think people risk harming themselves if they use each of the tobacco products. The four remaining items on a 4-point Likert scale, from strongly disagree to strongly agree, present statements such as: "The following products increase the risk for medical problems such as reproductive problems, respiratory problems, or heart disease" [81].	0.786
Perceived risk of using cigars		0.766
Perceived risk of using smokeless		0.718
Perceived risk of using hookah		0.846
Perceived risk of using e-cigarettes		0.865
Perceived personal benefits of e-cigarettes	This measure includes ten items adapted from the decisional balance measure, on a 4-point Likert scale, asking about participants' beliefs regarding the self-benefits of vaping e-cigarettes (e.g., "I think using e-cigarettes/personal vaporizers helps me stay slim") [82]	0.947
Perceived general benefits of e-cigarettes	This measure includes nine items adapted from the decisional balance measure, on a 4-point Likert scale, asking about participants' beliefs regarding the general benefits of e-cigarettes (e.g., "I think young adults use e-cigarettes/personal vaporizers to stay slim") [83].	0.895
Perceived addictiveness of products	This measure includes nine items on participants' perceived addictiveness of nine tobacco products [10].	0.922
Perceived popularity of tobacco use	This measure includes nine items on participants' beliefs regarding social acceptability of tobacco use among people their age [10].	0.924
Susceptibility to use cigarettes	Susceptibility to use is a dichotomous index to identify adolescents who are predisposed to use tobacco. It is measured based on intention to use in the future and social influence [82].	-
Susceptibility to use cigars		-
Susceptibility to use smokeless		-
Susceptibility to use hookah		-
Susceptibility to use e-cigarettes		-

Note. Cronbach's alpha is presented for data from the baseline survey.

[a] Pearson correlation coefficient instead of Cronbach's alpha for variables measured with only 2 items. PC1: post-campaign 1; PC2: post-campaign 2. Data for the 7-day post-campaign 1 and post-campaign 2 has not yet been completed, and so Cronbach's alpha values are not available yet.
